# Managing the risks and benefits of clinical research in response to a pandemic

**DOI:** 10.1017/cts.2021.14

**Published:** 2021-03-30

**Authors:** Patrick A. Flume, Elie F. Berbari, Laura Viera, Rachel Hess, Janine Higgins, Jennifer Armstrong, Linda Rice, Laura True, Reza Shaker, John B. Buse, Reynold A. Panettieri

**Affiliations:** 1 Departments of Medicine and Pediatrics, Medical University of South Carolina, Charleston, SC, USA; 2 Department of Medicine, Mayo Clinic, Rochester, MN, USA; 3 Clinical Research Support Office, North Carolina Translational and Clinical Sciences Institute, University of North Carolina School of Medicine, Chapel Hill, NC, USA; 4 Departments of Medicine and Population Health Sciences, University of Utah, Salt Lake City, UT, USA; 5 Department of Pediatrics, University of Colorado Anschutz Medical Campus, Aurora, CO, USA; 6 Center for Clinical and Translational Science, University of Kentucky, Lexington, KY, USA; 7 Department of Medicine, Medical College of Wisconsin, Milwaukee, WI, USA; 8 Department of Medicine, Rutgers University, New Brunswick, NJ, USA

**Keywords:** Clinical research, translational research, COVID-19, safety, governance

## Abstract

**Introduction:**

The coronavirus disease 2019 (COVID-19) created major disruptions at academic centers and healthcare systems globally. Clinical and Translational Science Awards (CTSA) fund hubs supported by the National Center for Advancing Translational Sciences provideinfrastructure and leadership for clinical and translational research at manysuch institutions.

**Methods:**

We surveyed CTSA hubs and received responses from 94% of them regarding the impact of the pandemic and the processes employed for the protection of research personnel and participants with respect to the conduct of research, specifically for studies unrelated to COVID-19.

**Results:**

In this report, we describe the results of the survey findings in the context of the current understanding of disease transmission and mitigation techniques.

**Conclusions:**

We reflect on common practices and provide recommendations regarding lessons learned that will be relevant to future pandemics, particularly with regards to staging the cessation and resumption of research activities with an aim to keep the workforce, research participants, and our communities safe in future pandemics.

## Introduction

The coronavirus disease-2019 (COVID-19) pandemic created major disruptions to the work of colleges and universities across the world starting in January 2020. Worker safety is an ethical, as well as legal and regulatory requirement and a primary focus of campuses and healthcare systems. As such, institutions implemented tactics in response to the severe acute respiratory syndrome coronavirus 2 (SARS-CoV-2) threat to reduce the spread of infection and preserve resources. These included the initial closure of most research activities at many institutions, restriction of personnel on campus, and reallocation of resources to prepare for a surge in COVID-19 cases. In the absence of a coordinated national response, institutions across the US- created site-specific policies and procedures that prioritized which, and under what circumstances, research activities could continue. We sought to learn how research institutions managed these issues. We recognized that decisions would be specific to each academic culture and aligned with their corresponding health system, hospital, and broader university policies, as well as local, state, and federal regulations under the auspices of institutional integrity and risk management programs. By examining this diversity, we sought to identify better practices that could be learned and implemented in response to any future emergency.

## Methods

We contributed to a larger survey submitted to the network of centers supported by the National Center for Advancing Translational Science’s (NCATS) Clinical and Translational Science Awards (CTSA) program as these centers provide infrastructure for major universities across the USA and is likely engaged in devising and implementing policies. The survey is included as a supplemental table in the companion paper entitled “‘Re-engineering The Clinical Research Enterprise in Response to COVID-19: The Clinical Translational Science Award (CTSA) Experience and Proposed Playbook for Future Pandemics” [1]. The submitted questions for this summary were reviewed and approved by a steering committee, and then included in the overall survey constructed in REDCap. The survey was sent to each hub’s primary investigators with a request for completion within 2 weeks. We assume the data were compiled by appropriate members of each hub’s team.

For this summary, we focused our survey on the protection of research personnel and the conduct of research unrelated to the COVID-19 pandemic. Research related to the SARS-CoV-2 virus and its effects is expected to be subject to different considerations and decisions, which are discussed in an accompanying paper. The survey questions were developed by the authors to address key questions relevant to decision-making and implementation in response to the pandemic. The questions sought to determine the impact of COVID-19 on research activities, including protections for trainees, faculty, and staff. Those surveyed were asked about decisions regarding the prioritization of research activities and how those determinations were established and communicated. Assuming that most, if not all, centers had allowed resumption of some research activities, we queried the extent of resumption and how those decisions were made as of late October 2020. Understanding the basis of some decisions, such as whether to limit personal interactions or to preserve personal protective equipment (PPE), we asked questions related to the approval processes and monitoring of research reactivation activities. We also inquired about the role of the CTSA hubs in the decision-making and/or implementation. Finally, we inquired regarding key lessons learned from this experience, what procedures or policies will remain in place after the pandemic is over, what steps would be implemented again, and what would be done differently.

We received responses from 60 sites (94%). Not all questions were completed and percentages are provided as appropriate.

## Results

### General Methods to Manage Risk of Infection

Airborne transmission of respiratory viral illness occurs when an infectious person breathes, speaks, eats, coughs, or sneezes resulting in aerosolized particles containing the virus that subsequently encounter a susceptible person’s mucus membranes. Evidence using polymerase chain reaction for SARS-CoV-2 also shows that the virus can be detected on fomites and in human specimens including blood, feces, urine, and mucus.

Biosafety committees needed to establish policies and procedures as to how such specimens could be handled [2]. Additionally, many institutions required principal investigators to submit a risk mitigation plan based on guidance provided and required training for all research personnel. Accordingly, mitigating contact by reducing exposure to infected individuals, de-densifying the environment, using PPE, and fastidious cleaning served as mainstays in preventing the spread of COVID-19. These policies are summarized in Table 1.

**Table 1.** Policies relevant to COVID-19 reported in the Clinical and Translational Science Awards hub survey

Common requirements for research
Return-to-work plans implemented by principal investigator with institutional guidance
Included cleaning and sanitation plans if that was not managed centrally
Few sites discussed provisions for staff who are in high-risk categories
Many sites included reminder posters to provide visual reference of guidelines in research spaces
Daily symptom checks for staff
Some sites required central reporting via app
Social distancing of at least 6 feet
Mandatory masks
Some sites allow cloth
Most mandate procedure or surgical for clinical research
Some required masks even in private offices
Hand washing and hand sanitation were required
Some sites required COVID-19 training modules prior to return to work
PPE provision varied from central distribution to individual lab procurement
Some sites required SARS-CoV-2 testing of staff prior to return to work (after closure)
All sites encouraged telework when possible
Phased definitions of allowable research
How phases were categorized and what research was allowed varied among sites
Most sites discussed procedures for breaks (e.g., eating), including distance in common areas and preference for eating outdoors
**Clinical research**
Some sites require eye protection for clinical participant interaction
Clinical research was encouraged to be conducted remotely when possible
No site required universal testing of research subjects for COVID-19
**Lab-based research**
All sites had provisions for maintaining cell lines and caring for animals
Density of personnel in labs was decreased
Many sites required occupancy limits to be posted
Some sites required institutional spot checks for compliance
One site recommended creating schedules to cohort people (e.g., group A works Monday, Wednesday, and Friday; group B works Tuesday and Thursday)
**Trainees (graduate students and postdoctoral trainees)**
Some sites required voluntary or mandatory COVID-19 surveillance
Depending on site, trainees were prioritized or delayed in returning to the lab
Some sites listed a designated institutional official to address trainee concerns that were not able to be resolved after discussions with principal investigator or other departmental leadership
One site delayed matriculation for the biomedical first-year PhD class

#### Keeping infected persons away from the workplace

A key aspect of preventing the spread of an infection is to isolate infected persons from susceptible hosts. This requires an understanding of who might be infectious. Testing, contact tracing, and knowledge of the infectivity period are critical components of any successful mitigation approach. For COVID-19, symptoms typically manifest on average about 5 days after exposure, but can occur later. Among those with symptoms, 98% will occur within 12 days of exposure. Yet, median infectivity can precede symptoms by ∼2 days [3] and investigators estimate that about half of all new infections originate from pre-symptomatic individuals [4], and a significant proportion of individuals may be asymptomatic or weakly symptomatic and still spread SARS-CoV-2. Monitoring for symptoms, therefore, ignores the potential for pre-symptomatic individuals and 15–40% of individuals who will never develop symptoms to unknowingly transmit the virus. Quarantine is recommended for individuals with an active infection or following a high- risk exposure. Despite recommendations for a 14-day quarantine following an exposure, 98% of all infections develop symptoms within 12 days. This led the CDC to recommend shorter quarantines of 7–10 days, especially when coupled with testing at the end of the quarantine period (https://www.cdc.gov/coronavirus/2019-ncov/more/scientific-brief-options-to-reduce-quarantine.html) [5]. A quarantine of 1 week after exposure can mitigate 80% of secondary infections [6].

Initially, the focus at the institutions surveyed was on symptomatic individuals, and many institutions required daily symptom and temperature monitoring for all research personnel. Approximately 20% of institutions required mandatory or voluntary routine COVID-19 testing protocols in place for faculty, trainees, and staff. These range from twice weekly testing to random testing. Initially, some CTSA sites (n = 11, 18%) required evidence of a negative SARS-CoV-2 molecular test to ascertain lack of infectivity following an infection as a condition to return to work.

Subsequent molecular testing of individuals who are positive is not currently recommended as PCR testing after symptoms abate can remain positive for much longer than the duration of infectivity. Shedding of nonviable RNA may extend beyond the infectivity period typically lasting around 10 days, with 68% becoming negative by day 28 and 95% being negative by day 33 [7].

#### Maintaining physical distance between persons

Physical distancing (e.g., the six-foot rule) emerges from the observations that droplets generated by a cough travel up to 6 feet into the ambient air. Speech may also produce droplets with most falling within 3–6 feet of the source. Quiet breathing produces essentially no large droplets and instead is limited to fine aerosols with reduced viral load. Physical distancing can partially mitigate the risk posed by the accumulation of and dispersion of droplets, but this risk is also dependent upon exposure time, room occupancy, airflow details, and activity intensity [6].

Results from our survey showed that most institutions mandated some form of physical distancing protocol including required scheduling of laboratory and other research space to reduce the number of people present at any one time. While physical distance can decrease the risk of exposure to the virus, it is inadequate by itself and there is a need for other measures to mitigate risk.

#### Personal protective equipment (PPE)

Masks and eye protection provide simple barriers to droplet projectiles from speech and cough. Masks are variably permeable to particles and are most effective if an infected person wears a mask to reduce the risk to others [8,9]. In our survey, all institutions required research staff, patients, and visitors (when allowed) to wear masks in common areas, except for times when individuals were eating or drinking. Provision of PPE was guided by institutional policies and ranged from a central repository linked to health system PPE to individual laboratory responsibility.

#### Policies to protect trainees and staff

Trainees and staff are particularly vulnerable to their supervisors’ adherence, or lack thereof, to policies meant to ensure their safety. We found that most institutions instituted policies to ensure that trainees and staff availed themselves of infection mitigation practices. Institutions created standard definitions for jobs that had to be conducted on campus and asked that those not in these positions work remotely as much as practical. Approximately 10% of institutions prioritized graduate trainees and postdoctoral fellows return to research in order to keep them on track for graduation or promotion. An equal proportion of institutions required permission for graduate trainees to resume in-person research and one institution delayed the matriculation of the first-year PhD class in laboratory-based research disciplines. Leave policies were modified to allow people with COVID-19-like symptoms or exposures to work remotely or take time off without penalty. Approximately 15% of institutions created channels to allow trainees and staff to report concerns specific to pandemic-related issues to institutional leadership.

### How were practices altered to address the challenges of the COVID-19 pandemic?

**Fig. 1.**
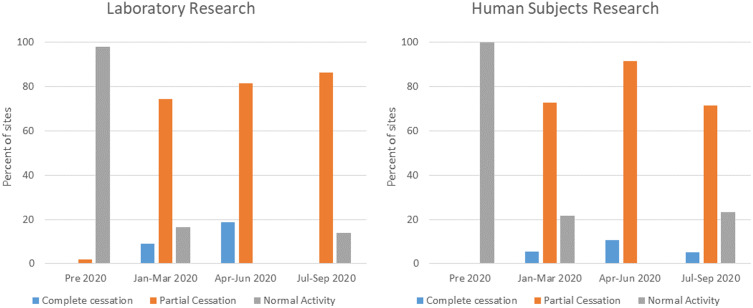
Cessation and resumption of research over time. Displayed are the percentage of Clinical and Translational Science Awards hub sites responding to the survey, which reported complete (blue) or partial (orange) cessation of research or normal research activities (grey) by time for laboratory-based research (left panel) and human subjects research (right panel).

Across the USA, there was geographic heterogeneity in the incidence, severity, and impact of COVID-19. While the East and West coast sites were first affected in the early spring of 2020, the Sun Belt states experienced a significant surge of COVID-19 in the summer, and the Midwest followed in the fall. Yet, the impact on research activities occurred early for all sites with cessation of laboratory and human subjects research in most by March 2020 (Fig. 1). These decisions were principally made by institutional leadership, such as the President, Provost, or leadership from the Office of Research, but only one site reported the decision had been made by the government (state and city) mandate. Eighty-six percent of CTSA hubs were involved to some extent in developing the institutional COVID-19-related policies. This included planning and/or implementation of shutting down of research activities (and subsequent reopening), primarily for clinical research with lesser involvement for development and implementation of policies for basic research across all sites (Table 2).

**Table 2.** Lessons learned from the pandemic related to prioritization of research

Implemented practices likely to remain in place after the pandemic
Remote research activities
Site monitoring
E-consenting
Virtual regulatory binders
Use of telemedicine for research
Mobile health tools
Remote collection of samples and data
Drive by study visits for essential safety labs and dispensing study medications
Shipping investigational product to clinical trial participants
Teleworking
Understand what work can be done remotely and how to support it
Learn how to monitor productivity
Practices that should be implemented again during another emergency
Centralized decision-making and guidance
Early convening of a decision-making committee
Engagement of central offices (e.g., Institutional Review Board) and stakeholders
Uniform adoption of safety procedures (e.g., facemasks)
Approval process for continuing or restarting research activities
Central communication
Coordinated
Broad reach
Nonpartisan
Frequent (no such thing as too much)
Robust support for remote research activities
Stockpiling of personal protective equipment and ensuring equitable and appropriate distribution
Prioritize resources to facilitate research directly relevant to the emergency

As few to no guidelines existed on how to manage research operations during a pandemic, sites developed their own protocols and operating procedures, nimbly revising them to address local Clinical and Translational Research (CTR) needs. For example, at the onset of the pandemic, and in anticipation of a regional or national surge in COVID-19 cases, research operations generally halted or minimized their activities (Fig. 1). Decisions were based on limiting the spread of SARS-CoV-2 among stakeholders (trainees, research personnel, healthcare workers, and study participants), maintaining critical aspects of research (e.g., sustaining key laboratory resources or continuing life-saving clinical trials), and preservation of PPE. Centers established task forces and initiatives to guide, prepare, and support the healthcare institutions and research operations during an unprecedented crisis [10] with a goal to minimize the disruption imposed by the pandemic and to provide a swift and safe reactivation of research operations. While non-COVID-19 research paused, centers pivoted to implement COVID-19-specific studies [11].

**Fig. 2.**
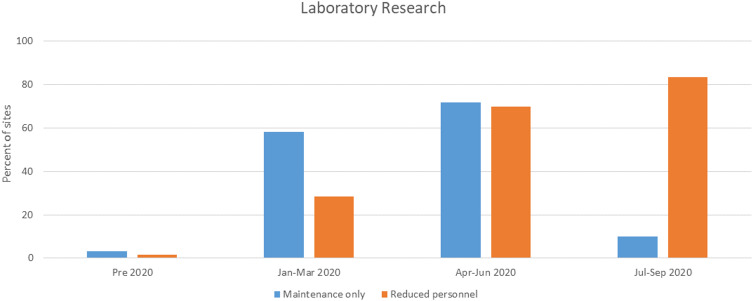
Permitted laboratory research activities. Displayed are the percentage of Clinical and Translational Science Awards hub sites responding to the survey, which reported only allowing maintenance activities (blue) or reduced personnel (orange) by time.

**Fig. 3.**
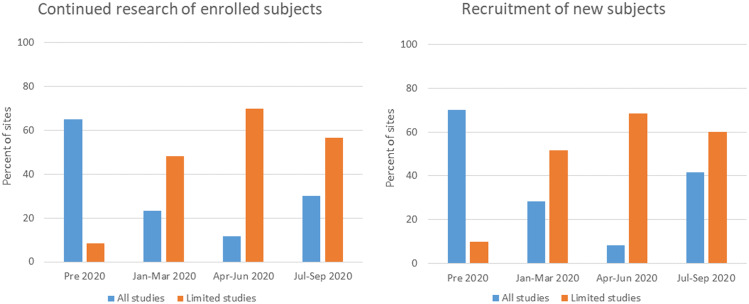
Permitted human subjects research activities. Displayed are the percentage of Clinical and Translational Science Awards hub sites responding to the survey, which reported all (blue) or limited (orange) clinical research activities with respect to continued research of enrolled subjects (left panel) or recruitment of new subjects (right panel) by time.

To reduce the number of persons on campus, most sites implemented policies that encouraged remote work when practical. All sites asked the persons to not come to campus if they were sick and most instituted some method of screening for symptoms of infection ranging from required self-monitoring to daily symptom reporting. For those sites that allowed some research operations, decisions were made to define and permit essential tasks only. Laboratory research was initially limited to tasks that were for maintenance only (e.g., preservation of cell lines and animal colonies) with no new experiments (Fig. 2). Human subjects research was rapidly reduced with nearly half of all sites (48%) converting active trials to remote activity only by the end of March. However, many sites permitted exceptions for some studies to continue with currently enrolled participants as well as new recruitment in the early stages of the pandemic (Fig. 3). These were typically described as interventional trials deemed to be potentially life-preserving (e.g., cancer therapies). Some sites permitted ongoing research if the activities were directly linked to the standard-of-care procedures (e.g., inpatient trials), were performed completely within the context of clinical care, or did not require face-to-face participant visits.

Institutions used a variety of techniques to communicate these rapidly evolving policies to their constituencies. Thirty-six percent conducted workshops, 83% conducted remote town halls, and all used email. Other methods included intranet portals dedicated to COVID-19 policies, videos, physical signs in buildings, and communication through monthly leadership meetings.

#### A phased reopening

In late October 2020, CTSA hubs generally noted a resumption of research activities (Figs. 1–3) that suggest a gradual return towards normalcy, but with only a minority describing pre-pandemic levels of operations. Nearly, all sites (90%) described a formal approval process for the resumption of research activities. In general, these requirements included limiting personnel and ensuring sufficient PPE, while some sites (n = 11, 18.3%) required negative testing for SARS-CoV-2 for personnel. Others require only symptom reporting and self-quarantine; there were some sites that collected such information while others depended on the honor system (i.e., self-monitoring and reporting).

Nearly, all CTSA hubs restarted CTR using a staged approach. These stages had specific goals and milestones that also focused on gradually repopulating personnel in labs and clinical facilities to respect social distancing rules. Despite the enormous burden on human and financial resources, the pandemic has provided an opportunity to redesign CTR at academic medical centers (AMCs). For example, electronic case report forms and consents are now standard operating procedures while regulatory processes have been streamlined and harmonized to maximize productivity and improve safety [12,13].

## Discussion

In response to the need to reduce morbidity and mortality related to the pandemic, CTSA institutions implemented a variety of steps intended to reduce the risk of transmission of infection. As expected there were many similarities, but some interesting differences, and there are some observations that may influence decision-making for similar circumstances in the future. Consistent with this, there were many lessons learned and reported by the sites. Many actions taken by sites are likely to be adopted as part of usual practice, and there were many steps that worked well and would be advised for implementation in the setting of another emergency (Table 2). Key in these measures is the need for centralized decision-making based upon engagement with stakeholders and institutional offices with clear communication to all stakeholders. Particularly for clinical trials and patient-facing research activities, aligning the healthcare system and university regulations and workflows is essential. For example, most of the institutions resumed elective surgery and procedures (e.g., colonoscopy and cardiac catheterization), but required SARS-CoV-2 testing prior to the procedure. Parallel policies were adopted by many institutions for clinical research that involved anesthesia or prolonged close contact between research staff and participants. Oversight, review, and approval to restart follow-up activities and new enrollment in clinical trials mitigates the inherent conflict of interest of investigators and protects trainees, staff, and participants. Coordinated communication pretested with stakeholders minimizes confusion and duplication. For future events, better preparation would include sufficient stockpiles of PPE, appropriate distribution and supply chain channels, and robust electronic platforms that allow work to continue remotely. Plans must be dynamic and nimble to adapt to changing conditions. Research that directly relates to the emergency and that which is therapeutically beneficial to patients must be prioritized and institutional resources committed to continue these activities. Policies must be in place that protects trainees and staff – allowing them to keep their positions while protecting their safety – free from retribution.

Several hubs also noted that action could have been taken in a more deliberate and moderated manner, rather than with the urgency in which it occurred. Better understanding of the “challenge” (for SARS-CoV-2, a largely droplet borne respiratory virus), the effectiveness of risk mitigation strategies, and potential weak links would have minimized fear and disruption. Greater understanding of the dynamics of disease transmission [6] for a particular site, region, or locale would have allowed sites to be more deliberate in their steps without unnecessarily stopping research activities. With greater knowledge of the level of endemic infection, administrators could enforce remediation measures as deemed feasible, and then increase or decrease safety measures according to the dynamic change in local transmission rates.

CTSA hubs played a key role at most institutions, and those institutions nimbly revised procedures and policies. Remote work environments, electronic consent and follow-up, and remote monitoring of studies were implemented. New platforms were deployed to support this work and personnel were rapidly trained in their use. Translational science moved promising therapeutics from bench to bedside at previously unheard of speed. Researchers and clinicians came together to move efficiently between clinical observation and mechanistic research, as well as between laboratory study and clinical testing. Both in our successes and stumbles, the responsiveness, coordination, and performance within our institutions to mitigate the impact of the pandemic has put us in a better position to deal with future emergencies. Infrastructure in the form of hardware, software, policies, procedures as well as the administrative processes endures. With time, there will be greater clarity about what worked well and what could be done better. Future research will further inform our responses to future challenges.

**Fig. 4.**
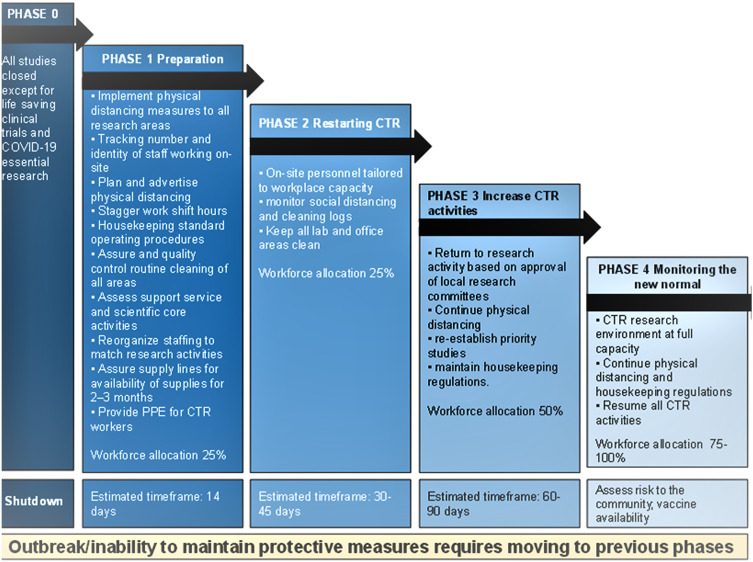
Phases of Clinical and Translational Research: The Renewal. The COVID-19 pandemic profoundly affected Clinical and Translational Research (CTR) in academic centers. In future emergencies, a phased approach, which mirrors common best practice during the current pandemic should be developed to protect the workforce, trainees, research participants, and the community while aiming to reconstruct CTR. Phase 0 represents a virtual shutdown of all CTR activities except those that can be performed remotely (“dry-lab” services) are mission-critical COVID-19 research, or provide access to important therapies. Nonessential personnel such as administrators, financial, and regulatory personnel work from home. In Phase 1, the facility/center is reconfigured to protect study participants and CTR personnel. Housekeeping standards and operations are defined to decrease fomite exposure, social distancing practices, and reminders are posted, staggered personnel shifts are devised, and personal protective equipment (PPE) supply chains are established. Phase 1 limits research staffing to 25% of the workforce. Phase 2 focuses on measuring the success of protective measures and the infectivity rates in the workforce implementing these precautions. Governance committees at the highest level establish policies implemented at the departmental level to triage and prioritize CTR projects for reopening. When clinical services resume, those projects that leverage routine visits without the need for additional interventions generally could resume. Phase 2 increases staffing to 50% of the workforce. Phase 3 incrementally increases CTR activities as possible based on stakeholder acceptance and community safety. Phase 3 studies are prioritized by clinical importance, institutional priorities, financial consequences, and opportunities to foster career development and training. Phase 3 allows 75% of the workforce to practice effective infection control as determined by measuring R_site_ v R_comm_. Phase 4 enables all CTR activities to resume. Figure modified from version in Nayeri et al. [14].

The history of the 1918 flu pandemic taught valuable lessons about an ordered approach [6]. Clear communication and transparency support compliance with pandemic policies, perseverance with risk mitigation steps avoid the consequences of a premature cessation of restrictions, and a phased reopening is recommended (Fig. 4) [14]. Despite a lack of a coordinated national response, AMCs adopted similar approaches to managing research programs with the goal of keeping our workforce and research participants safe by reducing the spread of SARS-CoV-2. CTSA hubs were involved in decision-making and implementation of measures in the cessation and eventual resumption of research activities. Priorities for non-COVID-19- related research were generally based on what was deemed to be essential for both laboratory (e.g., preserving cell lines) and human subjects (e.g., life-saving interventions) research. Sites adjusted their measures as more was learned. Many of these innovations, especially those that enabled remote research activities, are likely to remain a core aspect of clinical and translational research for the future. Ensuring an adequate supply of PPE and cleaning supplies is the key lesson to enhance safety in a future pandemic; anecdotally, COVID-19 spread among workers and research participants has been negligible since adequate supplies were available. Central decision-making with clear lines of authority and oversight to implement policies at the level of departments and individual research groups and a coordinated communication system is essential for optimal responses to future emergencies, including renewed surges of COVID-19.
